# Metagenome-assembled genomes of anelloviruses in crowned lemur and aye-aye swabs

**DOI:** 10.1128/mra.01473-25

**Published:** 2026-02-18

**Authors:** Elise N. Paietta, Simona Kraberger, Miriam Gordon, Erin Ehmke, Anne D. Yoder, Arvind Varsani

**Affiliations:** 1 The Biodesign Center for Fundamental and Applied Microbiomics, Center for Evolution and Medicine, School of Life Sciences, Arizona State University 7864, Tempe, Arizona, USA; 2 Department of Biology, Duke University 3065, Durham, North Carolina, USA; 3 Duke Lemur Center, Duke University 3065, Durham, North Carolina, USA; 4 Structural Biology Research Unit, Department of Integrative Biomedical Sciences, University of Cape Town 37716, Cape Town, South Africa; Portland State University, Portland, Oregon, USA

**Keywords:** crowned lemur, aye-aye, *Anelloviridae*

## Abstract

Two circular, complete genomes of anelloviruses were identified from a crowned lemur anal swab and an aye-aye skin swab from individuals at the Duke Lemur Center (Durham, NC, USA). The anelloviruses represent two species in the *Anelloviridae* family and expand a developing lemur-associated anellovirus lineage.

## ANNOUNCEMENT

Viruses in the *Anelloviridae* family have circular, single-stranded DNA genomes. Anelloviruses are considered ubiquitous and commensal with their vertebrate hosts (1, 2). Previous studies on anelloviruses in 3 out of more than 100 lemur species have provided preliminary evidence for the divergence of lemur anelloviruses from other known primate-associated anellovirus lineages (3, 4). Here, we investigate samples from captive crowned lemurs (*Eulemur coronatus*, family Lemuridae) and aye-ayes (*Daubentonia madagascariensis,* family Daubentoniidae) to expand our understanding of anellovirus diversity in lemurs. Given that anelloviruses are thought to co-evolve with their hosts, aye-ayes are of particular interest as they represent the most basal taxon of the lemuriform primates (5, 6).

**Fig 1:**
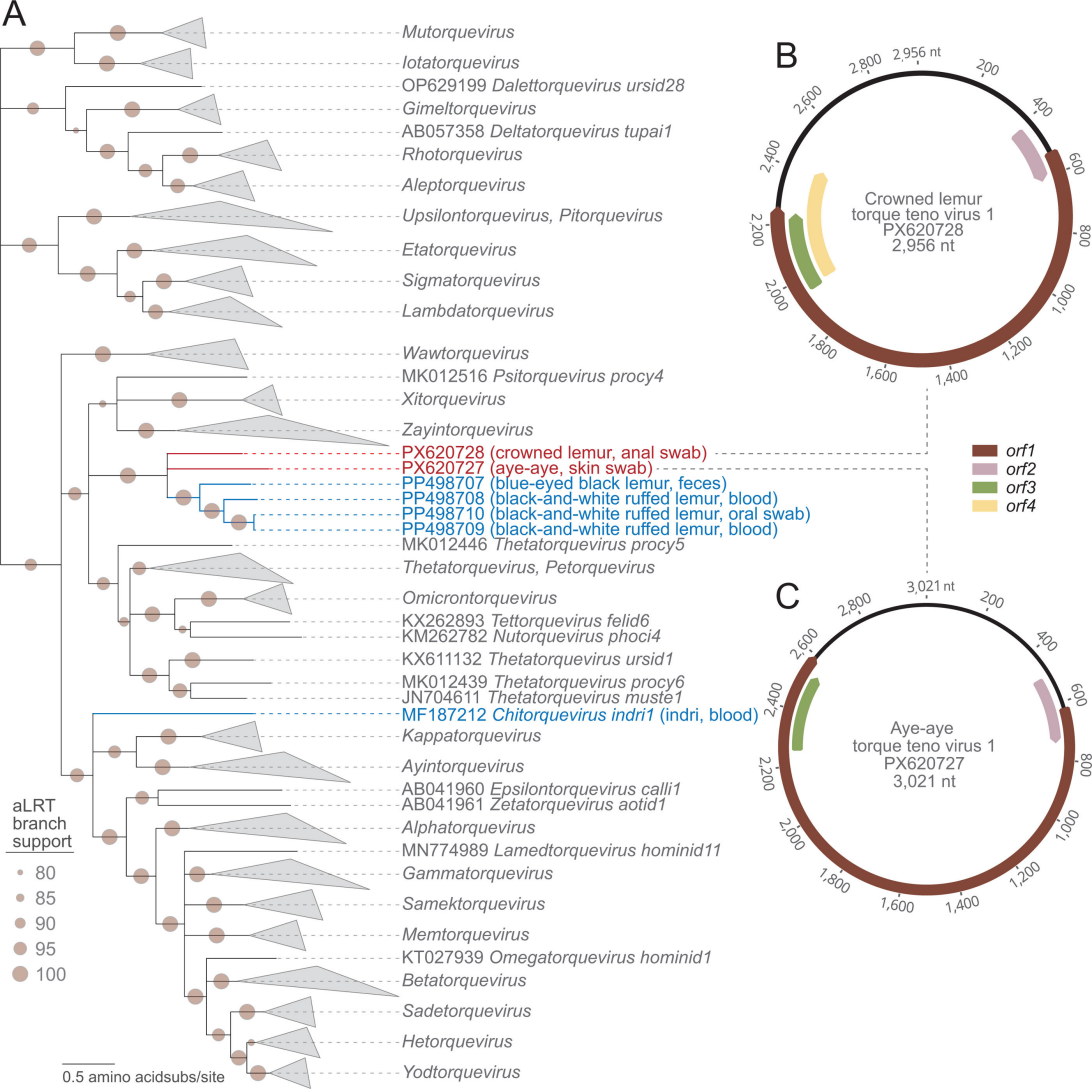
(**A**) Maximum likelihood phylogenetic tree of ORF1 amino acid sequences from anelloviruses described in this study, previously described lemur-derived anelloviruses, and established anellovirus species. Sequences identified here are shown in red font, while all previously described lemur-derived anelloviruses are shown in blue font. Branches with <0.8 aLRT were collapsed using TreeGraph (13), and the tree was rooted with gyrovirus ORF1 sequences. (**B**) Genome organization of crowned lemur torque teno virus 1. (**C**) Genome organization of aye-aye torque teno virus 1.

An anal swab from a crowned lemur was collected in June 2023, and a vulval skin swab of an aye-aye was collected in December 2024 from the Duke Lemur Center (Durham, NC, USA) under IACUC #A109-20-05. The swabs were stored in Universal Transport Media (Puritan, USA) and frozen at −80°C. DNA was extracted from each sample with the Roche High Pure Viral Nucleic Acid Kit (Roche Diagnostics, Germany). Circular DNA was then amplified using the Illustra Templiphi rolling circle amplification kit (GE Healthcare, USA). Libraries were generated with the Illumina DNA Prep Kit and sequenced on an Illumina NovaSeq X Plus at Psomagen Inc. Paired-end reads (2 × 150 bp) were trimmed with Trimmomatic v0.39 (7). Contigs were *de novo* assembled with MEGAHIT v1.2.9 (8), and circular contigs were tagged based on terminal redundancy. Genomes were annotated using CenoteTaker3 (9, 10) and supplemented with manual curation. Pairwise identities were calculated using SDT v1.2 (11). All bioinformatic tools were used with default settings. ORF1 sequences from anelloviruses identified here and in previous studies, along with all established anellovirus sequences, were aligned. A maximum likelihood phylogenetic tree of the ORF1 amino acid sequences was constructed with IQ-Tree2 (12) with the model finder option (best-fit model VT+F+R8) (Fig. 1A).

The aye-aye anellovirus genome (aye-aye torque teno virus 1; accession no. PX620727) is 3,021 nt in length (Table 1; Fig. 1C). The crowned lemur anellovirus genome (crowned lemur torque teno virus 1; accession no. PX620728) is 2,956 nt in length (Table 1; Fig. 1B). Aye-aye TTV-1 shares the highest *orf1* nucleotide identity of 56.4% with crowned lemur TTV-1. Crowned lemur TTV-1 shares 58.9% *orf1* nucleotide identity with a black-and-white ruffed lemur anellovirus (PP498708) and 56.0% with a blue-eyed black lemur (*Eulemur flavifrons*) anellovirus (PP498707), the only other anellovirus available from the *Eulemur* genus. Given that these share <69% pairwise similarity with other anellovirus *orf1* sequences (1), these tentatively represent two novel species. Phylogenetically, the ORF1 proteins cluster with the other previously identified lemur-derived anelloviruses and likely represent a new (unclassified) genus (Fig. 1A). These anelloviruses provide key information on anellovirus divergence in lemurs, not only for another species in *Eulemur* but for the only extant species in the divergent lemur family Daubentoniidae. This further supports a co-evolution of anelloviruses in lemurs, which have remained isolated in Madagascar from other non-human primates for *ca*. 65 My.

**TABLE 1:** Overview of anellovirus genomes identified in the *de novo* assemblies of vulval skin swab (21,226,296 paired reads; 15,076 contigs; N50: 1,586) of *Daubentonia madagascariensis* and anal swab (27,629,568 paired reads; 144,032 contigs; N50: 799) of *Eulemur coronatus*

	Accession	
	PX620727	PX620728
Virus	Aye-aye torque teno virus 1	Crowned lemur torque teno virus 1
Source sample type	Vulval skin swab	Anal swab
Source species	*Daubentonia madagascariensis*	*Eulemur coronatus*
Genome length	3,021 nt	2,956 nt
Topology, completeness	Circular, complete	Circular, complete
GC content	57.2%	50.2%
Mean coverage depth	1,389	26

## Data Availability

The two genomes described here have been deposited in GenBank under accession nos. PX620727 for aye-aye torque teno virus 1 and PX620728 for crowned lemur torque teno virus 1. The raw reads for these samples are deposited in SRA under BioProject no. PRJNA956591; Biosample nos. SAMN50457479 and SAMN53213548; SRA accession nos. SRR34868643 and SRR36075123.
